# Type III aortic arch angulation increases aortic stiffness: Analysis from an ex vivo porcine model

**DOI:** 10.1016/j.xjon.2023.10.035

**Published:** 2023-11-21

**Authors:** Tim J. Mandigers, Ariel F. Pascaner, Michele Conti, Martina Schembri, Sonja Jelic, Alessandra Favilli, Daniele Bissacco, Maurizio Domanin, Joost A. van Herwaarden, Ferdinando Auricchio, Santi Trimarchi

**Affiliations:** a Section of Vascular Surgery, Cardio Thoracic Vascular Department, Fondazione IRCCS Cà Granda Ospedale Maggiore Policlinico, Milan, Italy; b Department of Vascular Surgery, University Medical Centre Utrecht, Utrecht, The Netherlands; c Civil Engineering and Architecture Department, Università degli Studi di Pavia, Pavia, Italy; d Veterinary and Food Safety of Animal Origin Department, ATS Pavia, Pavia, Italy; e Department of Clinical Sciences and Community Health, Università degli Studi di Milano, Milan, Italy

**Keywords:** arch angulation, type III aortic arch, pulse wave velocity, aortic flow dynamics, TEVAR

## Abstract

**Objective:**

The relationship among increased aortic arch angulation, aortic flow dynamics, and vessel wall stiffness remains unclear. This experimental ex vivo study investigated how increased aortic arch angulation affects aortic stiffness and stent-graft induced aortic stiffening, assessed by pulse wave velocity (PWV).

**Methods:**

Porcine thoracic aortas were connected to a circulatory mock loop in a Type I and Type III aortic arch configuration. Baseline characteristics and blood pressures were measured. Proximal and distal flow curves were acquired to calculate PWV in both arch configurations. After that, a thoracic stent-graft (VAMF2626C100TU) was deployed in aortas with adequate proximal landing zone diameters to reach 10% t0 20% oversizing. Acquisitions were repeated for both arch configurations after stent-graft deployment.

**Results:**

Twenty-four aortas were harvested, surgically prepared, and mounted. Cardiac output was kept constant for both arch configurations (Type I: 4.74 ± 0.40 and Type III: 4.72 ± 0.38 L/minute; *P* = .703). Compared with a Type I arch, aortic PWV increased significantly in the Type III arch (3.53 ± 0.40 vs 3.83 ± 0.40 m/second; *P* < .001), as well as blood pressures. A stent-graft was deployed in 15 aortas. After deployment, Type I arch PWV increased (3.55 ± 0.39 vs 3.81 ± 0.44 m/second; *P* < .001) and Type III arch PWV increased although not significantly (3.86 ± 0.42 vs 4.03 ± 0.46 m/second; *P* = .094). Type III arch PWV resulted the highest and significantly higher compared with the Type I arch after stent-graft deployment (3.81 ± 0.44 vs 4.03 ± 0.46 m/second; *P* = .023).

**Conclusions:**

Increased aortic arch angulation—as in a Type III arch—is associated with higher aortic PWV and blood pressures and this may negatively influence cardiovascular health.

## Graphical abstract

**Figure:**
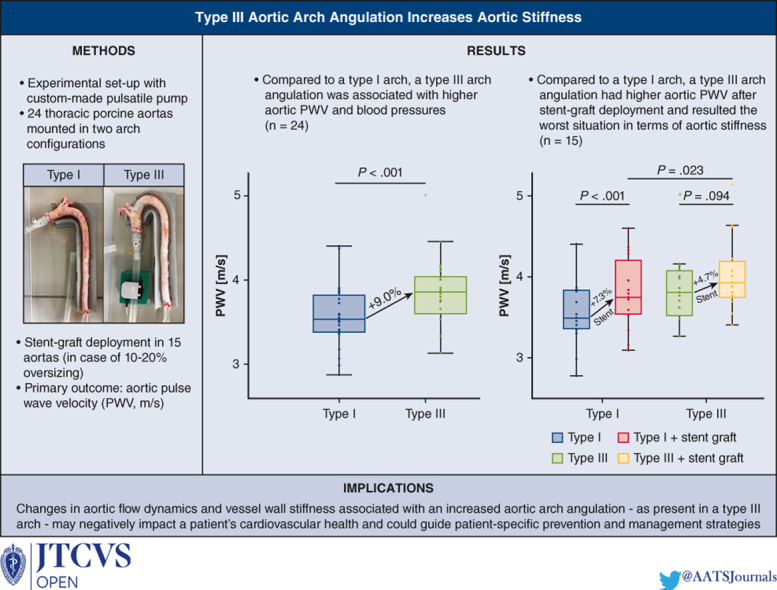

**Figure:**
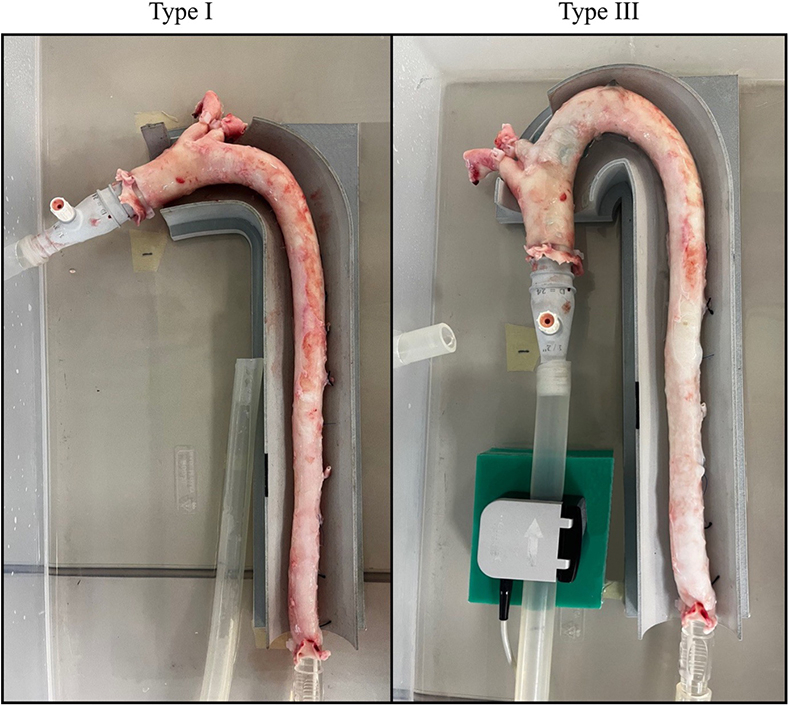
Thoracic porcine aortas in a Type I and Type III aortic arch configuration.

**Central Message**

Increased aortic arch angulation—as observed in a Type III arch—is associated with higher aortic PWV and blood pressures in this porcine ex vivo study.

**Perspective**

Previous literature has highlighted increases in systolic pulse wave reflections, central aortic stiffness, and hypertension in patients with postoperative geometrical configurations with an increased aortic arch angulation. The close relationship among these aspects remains to be further clarified and was studied by this experimental study utilizing a mock cardiovascular circulatory loop.

Successful thoracic endovascular aortic repair (TEVAR) of the aortic arch and proximal descending thoracic aorta is largely dependent on the anatomical characteristics of the landing zone of interest.^1^^,^^2^ The Modified Arch Landing Areas Nomenclature has characterized several geometric parameters associated with different aortic arch types in zones 0 through 3, and found increased angulation in the distal parts of the Type III arch.^3^ Such increased angulation has been associated with stent-graft malapposition, bird beak occurrence, and hostile hemodynamic displacement forces at the proximal landing zone (PLZ).^4^, ^5^, ^6^

In other postoperative geometrical configurations with an increased aortic arch angulation, like in patients who underwent successful open surgical repair of aortic coarctation or transposition of the great arteries, hypertension, increased systolic pulse wave reflections, and central aortic stiffness were also observed.^7^, ^8^, ^9^, ^10^ Aortic pulse wave velocity (PWV), widely adopted to quantify aortic stiffness, reflects the speed of pulse wave propagation along the aortic wall following left ventricular ejection, and independently predicts adverse cardiovascular events.^11^, ^12^, ^13^ Changes in aortic PWV have been investigated by numerous clinical and experimental studies after TEVAR, which showed that TEVAR increases aortic PWV.^11^^,^^14^, ^15^, ^16^, ^17^ Although the direct effect of TEVAR on aortic stiffening is known, the role of aortic arch angulation in this setting is less clear.

Therefore, this study aims to investigate the effect of an increased aortic arch angulation—as present in a Type III arch—on aortic PWV, using an ex vivo porcine model. The hypothesis was tested whether or not an angulated Type III arch, compared with a less angulated Type I arch, increases aortic PWV. Additionally, the study investigated if a Type III arch configuration influences TEVAR-induced aortic stiffening.

## Materials and Methods

Although the experimental setup of this study has been utilized to perform previous ex vivo analyses,^15^, ^16^, ^17^, ^18^, ^19^ several components (eg, ventricular compliance and transit-time derivation from flow curves) and protocol steps (eg, experiments within 12 hours of aortic sample procurement) have been updated, as described in detail below. The experimental protocol has not been previously published.

### Aortic Samples

Thoracic aortas of young healthy pigs (commercial hybrid, aged 10-12 months, weighing 160-180 kg) were procured at a local slaughterhouse from the ascending aorta to the level of the renal arteries. The pigs were evaluated by a veterinary physician, were solely raised for commercial purposes, and not killed for this study. Therefore, ethical approval by the local animal ethics committee was waived. The aortas were sealed and transported to the experimental β-lab of the University of Pavia. The experiments were performed on the same day, within 12 hours of procurement. The aortas were surgically prepared (T.J.M.) from the aortic root to the level of the celiac trunk by removing excess connective tissue and cardiac tissue. This allowed ligation of the 2 supra-aortic trunks, spinal arteries, and attachment of a proximal connector to the aortic root and a distal connector to the descending aorta.

### Experimental Setup and Components

**Figure 1:**
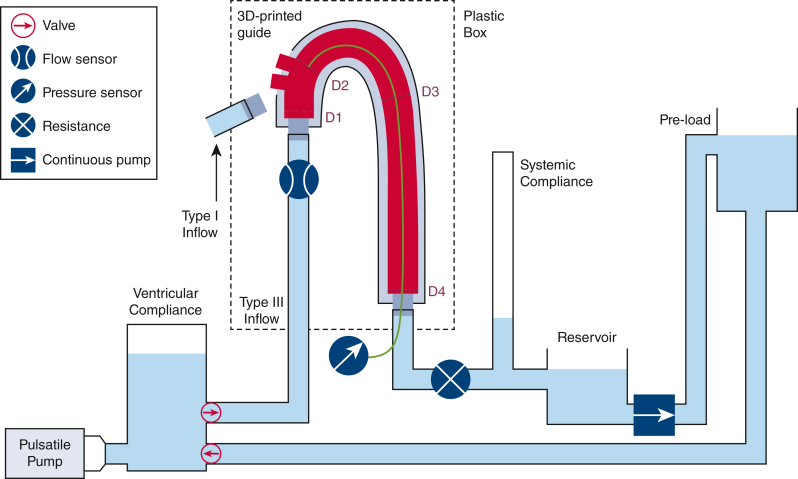
Schematic representation of the experimental setup and its components. *3D*, Three dimensional.

Figure 1 provides a schematic overview of the experimental setup and its components. The aortas were connected to a circulatory mock loop using silicone tubes and positioned in an open plastic box. The circulatory mock loop allowed for intraluminal pressurization under continuous steady state or pulsatile flow in a controlled manner. A centrifugal pump (Biomedicus 550; Medtronic) provided the continuous pressurization and a custom-made pulsatile pump resembling the left ventricle and containing both biomorphic inlet and outlet valves, provided pulsatile flow.^20^ A ventricular compliance has been added to this pulsatile pump to obtain stable pressure curves and to mitigate the high-frequency pressure variation due to the closure of the mechanical valves of the system. Water was utilized as circulatory fluid and kept at body temperature with a liquid heater (542 Heizer Titan [100 Watt]; Schego) in the water reservoir. Intraluminal pressures were recorded using a pressure sensor (40pc015g series; Honeywell) positioned in zone 3. Aortic flow was measured using a flow meter (Em·tec part of PSG, a Dover Company).

### Aortic Arch Guides

The aortas were guided into the desired aortic arch configuration utilizing 2 aortic guides with the geometrical characteristics of a Type I or a Type III aortic arch, as specified below. First, the aortic guides were virtually developed using computer-aided design. A basic virtual aortic model was created of which the geometry could be adjusted. According to baseline calibers of previously characterized thoracic porcine aortas (n = 20),^17^ mean aortic length, diameters at different points, and 2 supra-aortic trunks were inserted, so that the basic model virtually resembled a porcine thoracic aorta from the ascending aorta to the level of the celiac trunk.

Next, following the aortic arch classification, the basic virtual model was adjusted to create a Type I and Type III aortic arch model based on the vertical distance between the onset of the brachiocephalic trunk and the top of the aortic arch.^21^ Moreover, the geometric characteristics associated with a Type I or Type III aortic arch as defined by a previous study, were applied to both virtual models using similar methodology.^3^ These consisted of radius of curvature, aortic arch centerline length, tortuosity index, and ß-angle.

**Figure 2:**
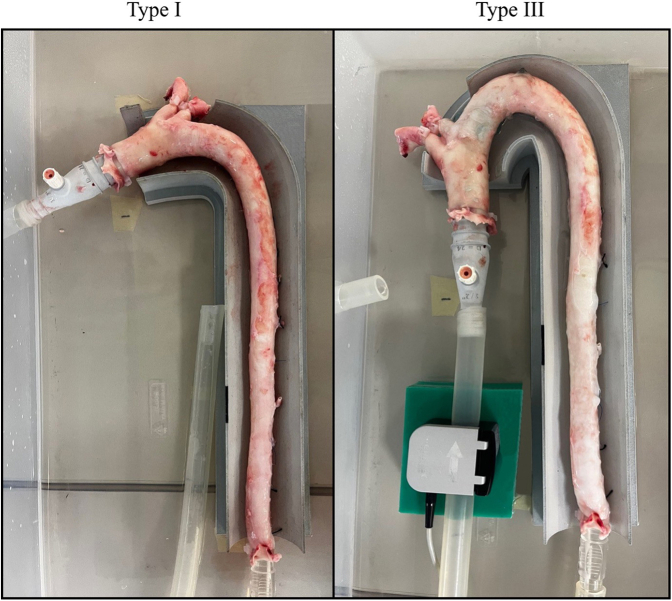
Thoracic porcine aortas with a Type I and Type III aortic arch configuration, connected to the experimental setup.

Both the Type I and III aortic arch guides were virtually designed around the Type I and Type III aortic models and a hatch was included for both supra-aortic trunks. The 2 virtual guides were consequently 3–dimensional-printed and utilized in the experimental setup (Figure 2).

### Experimental Workflow

The aorta was connected to the loop in the type I arch configuration and pressurized by continuous steady state flow. A planar image of the aorta was taken with a digital camera parallel to the aortic plane at arterial pressure levels of 80, 100, and 120 mm Hg to measure centerline length. At 100 mm Hg, baseline anteroposterior aortic diameters were measured using ultrasound (Accuvix XQ; Medison) by 2 operators (T.J.M. and A.F.P.) using an inner-to-inner calliper placement. Diameters were measured at 4 predefined locations: ascending aorta just distal to the proximal connector, just distal to the second supra-aortic trunk (ie, the PLZ), 112 mm distal to point 2 (ie, the distal landing zone), and descending aorta just before the distal connector (see dashed lines in Figure 1).

After baseline caliber measurements, pulsatile flow was installed and peripheral resistance, ventricular compliance, heart rate (75 beats per minute), and cardiac output (4.5-5.5 L/minute) were set to obtain physiologic baseline diastolic blood pressure (DBP), systolic blood pressure (SBP), pulse pressure (PP), and mean arterial pressure (MAP) values of 75 to 85, 115 to 125, 40 to 50, and 90 to 100 mm Hg, respectively. PP was defined as the difference between SBP and DBP.^22^ MAP was defined as DBP plus one-third of PP.^22^

Consequently, pressure values and aortic flow curves at the proximal and distal end of the aorta were acquired for at least 25 consecutive cardiac cycles. Next, the aorta was disconnected and guided into a Type III arch configuration utilizing the Type III arch guide. Planar images at the 3 MAP levels were retaken under continuous pressurization as in the Type I arch configuration. Then, the flow regime was changed to pulsatile and cardiac output (quantified as flow) was adjusted to achieve an equal cardiac output as in the Type I arch configuration if a flow reduction was noted. Here, the aim was to mimic physiologic compensation mechanisms of the heart with increases or decreases in pre- and/or afterload.^23^ Consequently, pressure values and proximal and distal aortic flow curves were acquired in the Type III arch configuration.

### Stent-Grafts and Implantation

A Valiant thoracic aortic stent-graft with the Captivia delivery system (Medtronic Inc) with a proximal and distal diameter of 26 mm and 112 mm covered length (Code: VAMF2626C100TU) was deployed in cases where an oversizing of 10% to 20% at the PLZ (just distal to the second supra-aortic trunk) could be achieved (following our stent-graft diameter, upper and lower cutoff aortic diameters to reach 10%-20% oversizing were 21.7-23.6 mm). A custom-made delivery system was utilized.^17^ Consequently, the stented aorta was reconnected to the circulatory loop, planar images, pressure values, and proximal and distal flow curves were acquired in both arch configurations, following the steps described above.

### Aortic PWV

**Figure E1:**
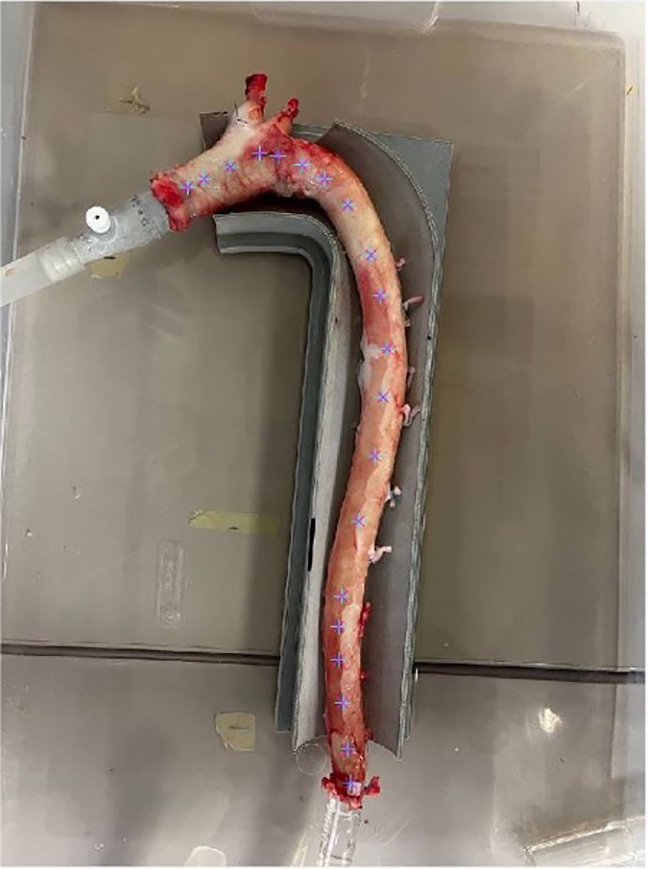
Illustration of operator-dependent manual placement of a minimum of 15 points between the proximal and distal connector to measure the centreline length of the aortic samples in Matlab (Mathworks).

Aortic PWV was calculated by dividing the centerline length of the aortic sample by the transit time over this distance. Transit time was obtained mechanically by applying the cross-correlation method^24^ between the proximal and distal flow curves, synchronized with the heart rate. Centerline length measurements were obtained by importing the planar images at different pressure values to Matlab (Mathworks), and manually placing a minimum of 15 points between the proximal and distal connector (A.F.P. and S.J.) (Figure E1). Pixels were scaled to centimeters using a reference line of 2.5 cm on the aortic guide. The change in length for different pressure levels and different arch configurations was accounted for: the length value used to compute aortic PWV was obtained by fitting a linear regression line between the pressure values at continuous flow and different length values. Consequently, length at the MAP levels during pulsatile flow was used to compute aortic PWV.

### Primary and Secondary Analyses

The primary analysis assessed changes in aortic PWV with increased aortic arch angulation (Type I vs Type III arch), without deployment of the stent-graft. The secondary analysis was the assessment of changes in aortic PWV for both arch configurations after stent-graft deployment and assessed whether an increased arch angulation affects TEVAR induced aortic stiffening.

### Sample Size Calculation

A power analysis was conducted based on a previous study that found a significant increase in aortic PWV in patients with an increased angulation of the aortic arch.^9^ With a 2-sided paired samples *t* test significance level of 5% (α = .050) and a power of 95%, the resulting required sample size was 10. To account for a potential margin of error, the number of experiments was set at a minimum of 15.

### Data Analysis

Data were analyzed using Microsoft Excel (Microsoft), Matlab version R2020b, and IBM SPSS Statistics version 28 (IBM-SPSS Inc). Data were reported as number and percentage, mean ± SD or median (interquartile range) where appropriate. Boxplots were created to graphically summarize results. Shapiro-Wilk test was performed to test for normality on all studied variables. Paired samples *t* test was performed to compare the means of 2 groups of normally distributed measurements and Wilcoxon signed rank test in case of nonnormally distributed data. A variability analysis was performed for the operator-dependent centerline length measurements, included in the calculation of aortic PWV. Intra- and interobserver reliability (ie, the extent to which the measurements can be replicated) was assessed for both arch configurations by calculating the intraclass correlation coefficient (ICC) (model: 2-way mixed, single rater/measurement, type: absolute agreement).^25^

## Results

### Baseline Aortic Sample Characteristics

A total of 24 aortas were harvested (November 2022-February 2023) and connected in both arch configurations for the primary analysis. In a subgroup of 15 aortas with an adequate diameter at point 2 (ranging from 21.7 to 23.6 mm) the stent-graft was deployed, and the aortas were connected in both arch configurations again for the secondary analysis. Table 1 reports baseline diameters and centerline length for the aortic samples. In the subgroup of 15 out of 24 (62.5%) aortas, PLZ oversizing at point 2 was controlled and was 14% ± 2%, which gradually increased toward the distal landing zone inherent to the tapering of the thoracic porcine aortas from proximal to distal aortic zones (Table 1).

**Table 1:** Baseline diameters and length of the thoracic aortic samples utilized for the primary and secondary analyses

Variable	Aortas for primary analysis (N = 24)	Subgroup of aortas for secondary analysis with stent-graft (n = 15)
Diameter point 1∗ (mm)	25.4 ± 1.8	25.3 ± 1.6
Diameter point 2∗ (mm)	22.8 ± 1.6	22.8 ± 0.5
Diameter point 3∗ (mm)	16.3 ± 1.0	16.2 ± 1.0
Diameter point 4∗ (mm)	14.6 ± 1.3	14.5 ± 1.3
Centreline length, Type I arch (cm)	36.2 ± 2.6	35.9 ± 3.1
Centreline length, Type III arch (cm)	36.9 ± 2.7	36.7 ± 3.0

Values are presented as mean ± SD.

∗ See the Materials and Methods section for a specification of the 4 diameter measurement locations.

### Primary Analysis

**Figure 3:**
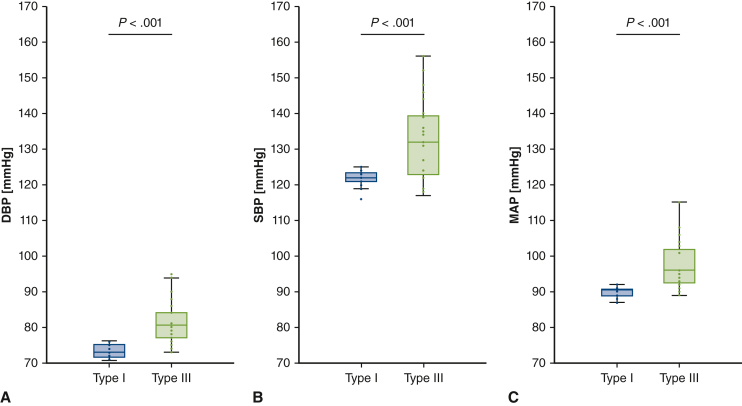
Boxplots of the diastolic (A), systolic (B), and mean arterial blood pressures (C) in both arch configurations for the 24 thoracic aortic samples. *Middle lines of the boxplots* represent median values. *Lower and upper border of the box* represent the 25th and 75th percentile (interquartile range), respectively. *Lower and upper whiskers* represent the minimum and maximum values of nonoutliers, respectively. *Points* represent individual data points and positive or negative outliers. *DBP*, Diastolic blood pressure; *SBP*, systolic blood pressure; *MAP*, mean arterial pressure.

**Figure 4:**
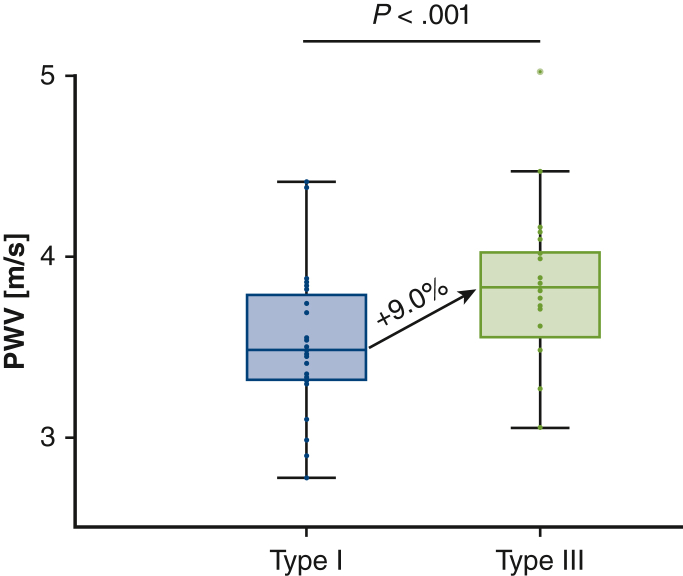
Boxplots of the aortic pulse wave velocity in both arch configurations for the 24 thoracic aortic samples. *Middle lines of the boxplots* represent median values. *Lower and upper border of the box* represent the 25th and 75th percentile (interquartile range), respectively. *Lower and upper whiskers* represent the minimum and maximum values of nonoutliers, respectively. *Points* represent individual data points and positive or negative outliers. *PWV*, Pulse wave velocity.

Table 2 reports the cardiac output, DBP, SBP, PP, MAP, and aortic PWV for both the Type I and Type III arch configuration. Cardiac output (flow), being a controlled parameter, was stable in both arch configurations (Type I: 4.74 ± 0.40 L/minute, Type III: 4.72 ± 0.38 L/minute; *P* = .703). With a change from Type I to Type III arch configuration, DBP, SBP, PP, and MAP significantly increased (Table 2). Figure 3 shows the changes in DBP, SBP, and MAP with respect to a change in arch configuration. Aortic PWV was significantly higher in the more angulated Type III arch with respect to the Type I configuration (Type I: 3.53 ± 0.40 m/second, Type III: 3.83 ± 0.40 m/second; *P* < .001), corresponding to a 9.0% ± 10% increase. Figure 4 shows the increase in aortic PWV with respect to a change in arch configuration for the 24 harvested aortas.

**Table 2:** Aortic flow, blood pressure, and pulse wave velocity values in both arch configurations for the primary and secondary analyses

Variable	Primary analysis (N = 24)			Subgroup of aortas for secondary analysis with stent-graft (n = 15)		
	Type I	Type III	*P* value	Type I	Type III	*P* value
Flow (L/min)	4.74 ± .40	4.72 ± .38	.703	4.70 ± .37	4.68 ± .33	.410
Diastolic blood pressure (mm Hg)	73 ± 2	81 ± 6	<.001	74 ± 3	77 ± 6	.030
Systolic blood pressure (mm Hg)	122 ± 2	133 ± 11	<.001	125 ± 6	133 ± 11	.002
Pulse pressure (mm Hg)	49 ± 2	51 ± 7	.024	51 ± 6	56 ± 8	<.001
Mean arterial pressure (mm Hg)	90 ± 1	98 ± 7	<.001	91 ± 3	96 ± 7	.008
Pulse wave velocity (m/sec)	3.53 ± .40	3.83 ± .40	<.001	3.81 ± .44	4.03 ± .46	.023

Values are presented as mean ± SD.

### Secondary Analysis

**Figure 5:**
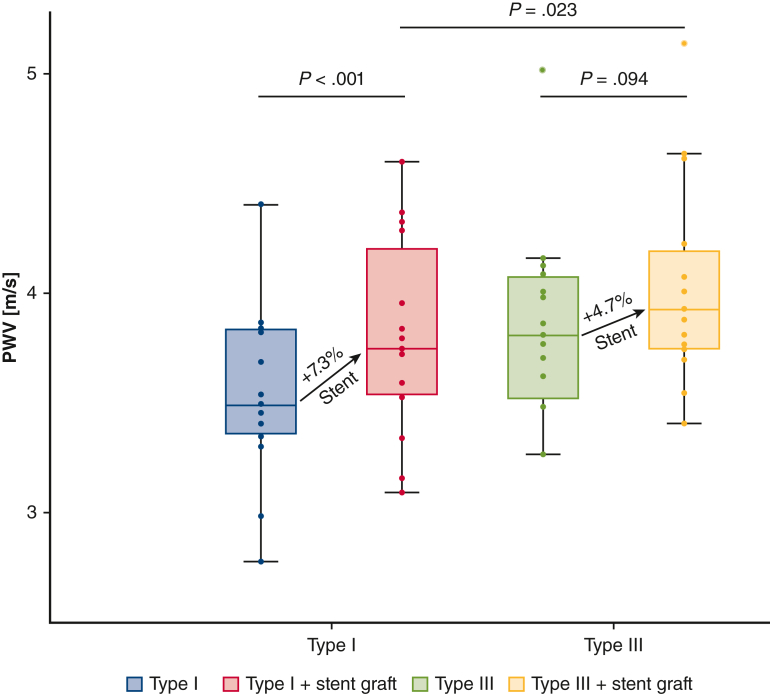
Boxplots of the aortic pulse wave velocity in both arch configurations before and after stent-graft deployment for 15 of the 24 thoracic aortic samples. *Middle lines of the boxplots* represent median values. *Lower and upper border of the box* represent the 25th and 75th percentile (interquartile range), respectively. *Lower and upper whiskers* represent the minimum and maximum values of nonoutliers, respectively. *Points* represent individual data points and positive or negative outliers. *PWV*, Pulse wave velocity.

In the subgroup of 15 aortas, there was an increase in aortic PWV after stent-graft deployment in the Type I arch (baseline PWV: 3.55 ± 0.39 m/second, PWV after TEVAR: 3.81 ± 0.44 m/second; *P* < .001). In the Type III arch, there was an increase in aortic PWV after stent-graft deployment; however, not statistically significant (baseline PWV: 3.86 ± 0.42 m/second, PWV after TEVAR: 4.03 ± 0.46 m/second; *P* = .094). As demonstrated in the primary analysis, the baseline aortic PWV before stent-graft deployment was higher in the Type III arch configuration compared with the Type I arch configuration. The mean percent TEVAR-induced increase in aortic PWV for the Type I arch configuration was 7.3% ± 5.3% and 4.7% ± 9.1% in the Type III arch configuration. Figure 5 shows the changes in aortic PWV for the subgroup of 15 aortic samples in which the stent-graft was deployed.

After stent-graft deployment, the increase in aortic PWV associated with a change to the Type III arch was lower compared with the primary analysis without stent-graft (6.4% ± 10% vs 9.0% ± 10%). Nevertheless, aortic PWV in the Type III arch after stent-graft deployment was highest and significantly higher compared with the Type I arch (Type I after TEVAR: 3.81 ± 0.44 m/second, Type III after TEVAR: 4.03 ± 0.46 m/second; *P* = .023) (Figure 5).

### Variability Analysis

In the Type I arch configuration, the intra- and interobserver ICCs of the centerline length measurements were 0.990 (95% CI, 0.978-0.996) and 0.990 (95% CI, 0.946-0.997). In the Type III arch configuration, the intra- and interobserver ICCs were 0.994 (95% CI, 0.985-0.997) and 0.978 (95% CI, 0.692-0.994), indicating excellent reliability for both arch configurations (ICC > 0.9).^25^

## Discussion

**Figure 6:**
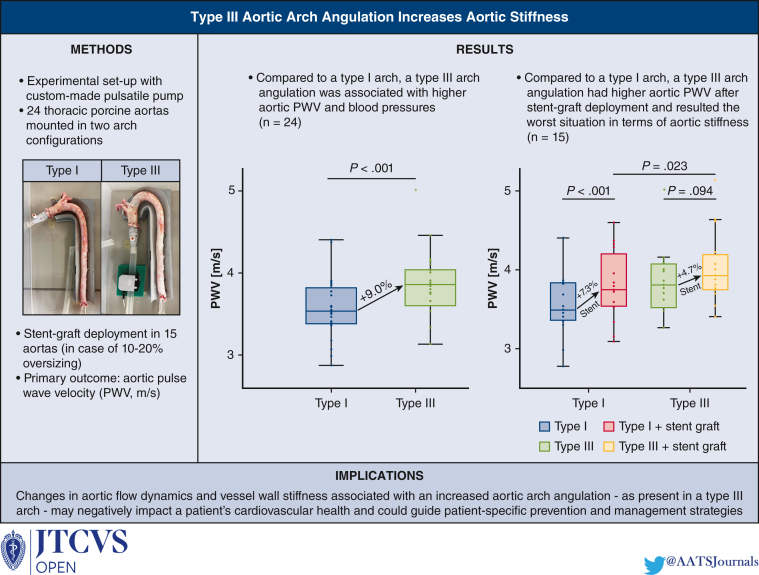
Type III aortic arch angulation increases aortic stiffness. *Middle lines of the boxplots* represent median values. *Lower and upper border of the box* represent the 25th and 75th percentile (interquartile range), respectively. *Lower and upper whiskers* represent the minimum and maximum values of nonoutliers, respectively. *Points* represent individual data points and positive or negative outliers. *PWV*, Pulse wave velocity.

The main findings of this study highlight significant changes in aortic flow dynamics and blood pressure responses following changes in aortic arch geometry (Figure 6). DBP, SBP, PP, and MAP increased with increasing arch angulation as in a Type III arch configuration, compared with a less angulated Type I arch. Moreover, aortic PWV increased in a Type III arch compared with a Type I arch. In addition, the study showed that Type III arch PWV is significantly higher compared with Type I arch PWV after stent-graft deployment. The study also confirms that thoracic stent-graft deployment increases aortic PWV.^11^^,^^14^, ^15^, ^16^ TEVAR in zone 3 of a Type I arch increased aortic PWV more than after TEVAR in zone 3 of a Type III arch, probably because aortic PWV was already significantly increased in the Type III arch configuration, as found in this study. These findings further underline that an increased arch angulation as in a Type III arch may be hostile, not only in terms of potential device-related complications, but also in terms of blood pressure responses, cardiac afterload, and aortic stiffness.^4^, ^5^, ^6^ Such changes in aortic flow dynamics could in turn be the cause of TEVAR failure. As an accepted surrogate for aortic stiffness, increases in aortic PWV result in an increased cardiac workload, pulsatile damage to target organs operating at high flow and low vascular resistance (eg, kidneys and brain), and could thereby negatively influence cardiovascular health.^11^, ^12^, ^13^, ^14^

Previous studies have demonstrated that different arch geometries (eg, gothic, crenel, romanesque) exist after successful open surgical repair of aortic coarctation, generally performed in young adults.^7^ These studies independently associated the gothic arch geometry with abnormal blood pressure responses, increased central aortic stiffness, and left ventricular mass, and highlighted the potential importance of aortic geometry on aortic flow dynamics.^7^, ^8^, ^9^ Gothic arch was defined as being acutely angulated between the ascending and descending aorta with a shortened or absent inner arch segment. The definitions of such arch geometries were however assessed globally on magnetic resonance imaging, compared with the clear definition of arch types (ie, Type I or Type III) based on multiple geometrical parameters in this study.^3^

A potential reason for the increases in blood pressure and aortic PWV with increases in arch angulation could be an increased systemic vascular resistance and thus cardiac afterload, resulting in compensation mechanisms that may increase mean blood pressure, and consequently aortic PWV. Although there were no structural changes to the aortic wall with changes in aortic arch type in this study, the highly nonlinear mechanical behavior of the aorta and the multiscale organization of lamellae, elastin, and collagen fiber of the aortic wall might result in a less efficient damping of the pulsatile propulsions during the cardiac cycle as MAPs increase.^26^ Moreover, it should be emphasized that this experimental setup utilized thoracic porcine aortas from healthy and young pigs. In patients with thoracic aortic disease, such aspects may even be more or less pronounced. This deserves further exploration to better understand the relationship between geometric arterial changes and blood pressure or aortic PWV responses.

Because a change in aortic geometry does not imply changes to the arterial wall, the validity of utilizing aortic PWV as a surrogate for aortic stiffness in this scenario could be debated. Namely, aortic PWV is dependent on Young's elastic modulus, thickness of the aortic wall, aortic radius, and fluid density following the Moens-Korteweg equation.^27^ Aortic PWV should thus not be interpreted as synonym of aortic wall elasticity because there is a complex interplay between Young's elastic modulus and geometric characteristics that play a major role in the estimation of PWV.^27^

### Future Perspectives

The results of ex vivo studies on aortic flow dynamics and the mechanical coupling between thoracic aortic stent-grafts and the native aorta could be compared with in-silico or in-vivo analyses to evaluate similarities and differences in findings. The development of a 3-dimensional, printable elastic aortic material strong enough to withstand pulsatile pressurization would allow the development of aortic models with specific geometries (eg, diameter, length, angulation, and tortuosity) with or without disease (eg, aneurysm). The addition of 4–dimensional-flow magnetic resonance imaging may provide additional insights into changes in aortic flow dynamics following changes in arch geometry.^28^

### Limitations

This study has limitations that are related to the experimental design and the use of porcine thoracic aortas, inherently limiting the translational value to human beings, and that must be acknowledged.^29^ Several aspects have been mentioned by previous studies utilizing this setup such as the use of water as perfusion fluid and the absence of surrounding tissue.^15^, ^16^, ^17^, ^18^, ^19^ The setup aims to isolate and analyze a specific parameter (eg, aortic PWV and blood pressure), whereas there is variability in other parameters at the same time (eg, aortic specimen diameters and length and distal oversizing). However, the experimental setup and systematic workflow allows us to control other factors (eg, baseline blood pressures, type of aortic arch, and proximal oversizing) to perform comparative analyses. In the secondary analysis, aortic sample selection bias to reach adequate oversizing might theoretically have influenced our findings. Reusing a single, nontapered, thoracic stent-graft did not result in macroscopic damage of the stent-graft.

## Conclusions

This porcine ex vivo study shows that an increased aortic arch angulation—as present in a Type III aortic arch—increases DBP, SBP, PP, MAP, and aortic PWV. This highlights that changes in arch geometry (eg, increased angulation) can result in altered aortic flow dynamics. Hypertension and aortic PWV, as a surrogate for aortic stiffness, increase a patient's cardiovascular risk. Future studies are needed to better explore the relationship between changes in aortic arch geometry, blood pressure response, and aortic stiffness, which might implicate changes in device materials and designs.

## Conflict of Interest Statement

Dr van Herwaarden is or has been a proctor or consultant for Gore Medical, Terumo Aortic, and Cook Medical. Dr Trimarchi is consultant and speaker for Medtronic Inc, WL Gore, and Terumo Aortic. All other authors reported no conflicts of interest.

The *Journal* policy requires editors and reviewers to disclose conflicts of interest and to decline handling manuscripts for which they may have a conflict of interest. The editors and reviewers of this article have no conflicts of interest.

## References

[1] Isselbacher E.M., Preventza O., Black J.H., Augoustides J.G., Beck A.W., Bolen M.A. (2022). 2022 ACC/AHA guideline for the diagnosis and management of aortic disease: a report of the American Heart Association/American College of Cardiology Joint Committee on clinical practice guidelines. Circulation.

[2] Czerny M., Schmidli J., Adler S., van den Berg J.C., Bertoglio L., Carrel T. (2019). Current options and recommendations for the treatment of thoracic aortic pathologies involving the aortic arch: an expert consensus document of the European Association for Cardio-Thoracic surgery (EACTS) and the European Society for Vascular Surgery (ESVS). Eur J Cardiothorac Surg.

[3] Marrocco-Trischitta M.M., de Beaufort H.W., Secchi F., van Bakel T.M., Ranucci M., van Herwaarden J.A. (2017). A geometric reappraisal of proximal landing zones for thoracic endovascular aortic repair according to aortic arch types. J Vasc Surg.

[4] Boufi M., Guivier-Curien C., Deplano V., Boiron O., Loundou A.D., Dona B. (2015). Risk factor analysis of bird beak occurrence after thoracic endovascular aortic repair. Eur J Vasc Endovasc Surg.

[5] Marrocco-Trischitta M.M., van Bakel T.M., Romarowski R.M., de Beaufort H.W., Conti M., van Herwaarden J.A. (2018). The Modified Arch Landing Areas Nomenclature (MALAN) improves prediction of stent graft displacement forces: proof of concept by computational fluid dynamics modelling. Eur J Vasc Endovasc Surg.

[6] Marrocco-Trischitta M.M., Romarowski R.M., De Beaufort H.W., Conti M., Vitale R., Secchi F. (2019). The Modified Arch Landing Areas Nomenclature identifies hostile zones for endograft deployment: a confirmatory biomechanical study in patients treated by thoracic endovascular aortic repair. Eur J Cardiothorac Surg.

[7] Ou P., Bonnet D., Auriacombe L., Pedroni E., Balleux F., Sidi D. (2004). Late systemic hypertension and aortic arch geometry after successful repair of coarctation of the aorta. Eur Heart J.

[8] Ou P., Mousseaux E., Celermajer D.S., Pedroni E., Vouhe P., Sidi D. (2006). Aortic arch shape deformation after coarctation surgery: effect on blood pressure response. J Thorac Cardiovasc Surg.

[9] Ou P., Celermajer D.S., Raisky O., Jolivet O., Buyens F., Herment A. (2008). Angular (Gothic) aortic arch leads to enhanced systolic wave reflection, central aortic stiffness, and increased left ventricular mass late after aortic coarctation repair: evaluation with magnetic resonance flow mapping. J Thorac Cardiovasc Surg.

[10] Agnoletti G., Ou P., Celermajer D.S., Boudjemline Y., Marini D., Bonnet D. (2008). Acute angulation of the aortic arch predisposes a patient to ascending aortic dilatation and aortic regurgitation late after the arterial switch operation for transposition of the great arteries. J Thorac Cardiovasc Surg.

[11] Bissacco D., Conti M., Domanin M., Bianchi D., Scudeller L., Mandigers T.J. (2022). Modifications in aortic stiffness after endovascular or open aortic repair: a systematic review and meta-analysis. Eur J Vasc Endovasc Surg.

[12] Chirinos J., Segers P., Hughes T., Townsend R. (2019). Large-artery stiffness in health and disease: JACC State-of-the-Art Review. J Am Coll Cardiol.

[13] Sutton-Tyrrell K., Najjar S.S., Boudreau R.M., Venkitachalam L., Kupelian V., Simonsick E.M. (2005). Elevated aortic pulse wave velocity, a marker of arterial stiffness, predicts cardiovascular events in well-functioning older adults. Circulation.

[14] Mandigers T.J., Bissacco D., Domanin M., D'Alessio I., Tolva V.S., Piffaretti G. (2022). Cardiac and aortic modifications after endovascular repair for blunt thoracic aortic injury: a systematic review. Eur J Vasc Endovasc Surg.

[15] de Beaufort H.W.L., Conti M., Kamman A.V., Nauta F.J.H., Lanzarone E., Moll F.L. (2017). Stent-graft deployment increases aortic stiffness in an ex vivo porcine model. Ann Vasc Surg.

[16] De Beaufort H.W.L., Coda M., Conti M., Van Bakel T.M.J., Nauta F.J.H., Lanzarone E. (2017). Changes in aortic pulse wave velocity of four thoracic aortic stent grafts in an ex vivo porcine model. PLoS One.

[17] Mandigers T.J., Conti M., Allievi S., Dedola F., Bissacco D., Bianchi D. (2023). Comparison of two generations of thoracic aortic stent grafts and their impact on aortic stiffness in an ex vivo porcine model. EJVES Vasc Forum.

[18] Nauta F.J.H., Conti M., Marconi S., Kamman A.V., Alaimo G., Morganti S. (2016). An experimental investigation of the impact of thoracic endovascular aortic repair on longitudinal strain. Eur J Cardiothorac Surg.

[19] Nauta F.J.H., De Beaufort H.W.L., Conti M., Marconi S., Kamman A.V., Ferrara A. (2017). Impact of thoracic endovascular aortic repair on radial strain in an ex vivo porcine model. Eur J Cardiothorac Surg.

[20] Lanzarone E., Vismara R., Fiore G.B. (2009). A new pulsatile volumetric device with biomorphic valves for the in vitro study of the cardiovascular system. Artif Organs.

[21] Madhwal S., Rajagopal V., Bhatt D.L., Bajzer C.T., Whitlow P., Kapadia S.R. (2008). Predictors of difficult carotid stenting as determined by aortic arch angiography. J Inv Cardiol.

[22] Vlachopoulos C., O'Rourke M., Nichols W.W. (2011).

[23] Bowman A.W., Kovács S.J. (2003). Assessment and consequences of the constant-volume attribute of the four-chambered heart. Am J Physiol Heart Circ Physiol.

[24] Fielden S.W., Fornwalt B.K., Jerosch-Herold M., Eisner R.L., Stillman A.E., Oshinski J.N. (2008). A new method for the determination of aortic pulse wave velocity using cross-correlation on 2D PCMR velocity data. J Magn Reson Imaging.

[25] Koo T.K., Li M.Y. (2016). A guideline of selecting and reporting intraclass correlation coefficients for reliability research. J Chiropr Med.

[26] Díaz C., Peña J.A., Martínez M.A., Peña E. (2021). Unraveling the multilayer mechanical response of aorta using layer-specific residual stresses and experimental properties. J Mech Behav Biomed Mater.

[27] Pascaner A.F., Houriez-Gombaud-Saintonge S., Craiem D., Gencer U., Casciaro M.E., Charpentier E. (2021). Comprehensive assessment of local and regional aortic stiffness in patients with tricuspid or bicuspid aortic valve aortopathy using magnetic resonance imaging. Int J Cardiol.

[28] de Beaufort H.W., Shah D.J., Patel A.P., Jackson M.S., Spinelli D., Yang E.Y. (2019). Four-dimensional flow cardiovascular magnetic resonance in aortic dissection: assessment in an ex vivo model and preliminary clinical experience. J Thorac Cardiovasc Surg.

[29] de Beaufort H., Ferrara A., Conti M., Moll F., van Herwaarden J., Figueroa C. (2018). Comparative analysis of porcine and human thoracic aortic stiffness. Eur J Vasc Endovasc Surg.

